# Procedures under tourniquet in sickle cell disease: safety evaluated in two hundred and thirty three sickle-cell disease anaemia adult patients in comparison with outcomes in five hundred and seventy four sickle cell anaemia patients with procedures without tourniquet

**DOI:** 10.1007/s00264-025-06510-7

**Published:** 2025-04-07

**Authors:** Philippe Hernigou

**Affiliations:** Paris-Est Créteil University, Créteil, France

**Keywords:** Sickle cell disease, Tourniquet, Transfusion, Blood loss

## Abstract

**Purpose:**

There is a lack of data evaluating the impact of tourniquet versus no tourniquet surgery in patients with sickle cell disease (SCD).

**Methods:**

The records of 233 sickle cell patients who underwent orthopaedic surgery with a tourniquet between 1978 and 2018 were retrospectively reviewed. This study group (233 patients) was compared to a control group of 574 SCD patients followed by the same surgical team in the same hospital undergoing the same procedures in the same period between 1978 and 2018 but without a tourniquet. Outcomes assessed skin complications, thrombophlebitis, bone necrosis, muscle necrosis or abnormal muscle function, peripheral nerve impairment, elevated blood pressure, post-operative sickle cell crises, and blood loss under a tourniquet.

**Results:**

The pneumatic tourniquet was primarily applied proximally in both lower and upper limbs. The median tourniquet duration was 65 minutes, with most procedures lasting between 30 and 90 minutes . Postoperative medical complications occurred in both groups, with no significant difference in hospital stay (6.7 vs. 7.1 days). Painful sickling crises affected 86 patients, with a lower prevalence in transfused patients (*p* = 0.04). Blood loss was significantly lower in the tourniquet group during knee surgeries (438 ml vs. 731 ml, *p* = 0.031), resulting in fewer transfusions. Skin complications did not affect wound healing. The 90-day incidence of venous thromboembolism (VTE) was 0.4%, with no significant difference between groups. Muscle biopsies showed no necrosis immediately post-surgery, but some necrosis appeared after 12 weeks in the tourniquet group. New bone osteonecrosis cases and infection rates were similar between groups.

**Conclusion:**

this study provides valuable insights into the use of tourniquets in sickle cell disease.

Sickle cell disease (SCD), the most common hereditary blood disorder [1], results from inheriting two abnormal haemoglobin genes. The disease is caused by a mutation that replaces glutamic acid with valine at the sixth position of the beta chain, leading to the formation of haemoglobin S (HbS). When deoxygenated, HbS polymerizes into long fibers, deforming red blood cells into a sickle shape. The balance between HbS in liquid and solid states depends on oxygen levels, HbS concentration, and other haemoglobins. Low blood pH and high HbS concentrations promote sickling. Deoxygenation also alters the red cell membrane, triggering clotting. Tourniquet use in surgery poses risks for sickle cell patients, as circulatory stasis, acidosis, and hypoxemia can exacerbate red cell deoxygenation and sickling, increasing the likelihood of complications. As of 2021, approximately 7.74 million people are living with SCD globally. Despite the prevalence of this condition, there are surprisingly few publications documenting orthopaedic surgeries performed using tourniquets in patients with SCD. This scarcity may stem from the limited experience of many surgeons with this pathology and/or the associated apprehension regarding tourniquet use in such cases.

The number of documented cases remains remarkably low across different regions. In 2017, Pignatti et al. [2] reported data on only 32 patients with Hb SS and six with Hb SC who had undergone surgery with a tourniquet. Furthermore, many of these publications [3] frequently conflate sickle cell anaemia with sickle cell trait, the latter being a genetic condition rather than a disease in most cases.

The author brings 50 years of experience performing surgeries with tourniquet use in sickle cell patients, beginning with the first case in 1974—an ankle fracture surgery in a patient with sickle cell anaemia in North Africa. More recent cases include total knee replacements performed under a tourniquet in patients with SCD. With a record of 1,500 sickle cell anemia patients operated on over five decades [4–5], the author reports his experience with tourniquet use in 233 patients with sickle cell anaemia. These findings are compared with outcomes from 574 sickle cell anaemia patients who underwent surgery without the use of a tourniquet.

## Materials and methods

Tourniquets are commonly used in limb surgeries, both on bone and soft tissue, to create a bloodless field, enhancing surgical accuracy, speed, and safety while reducing blood loss. A typical tourniquet system includes a pneumatic cuff placed proximal to the surgical site, and a pressure-maintaining mechanism. Before inflation, the limb is sometimes exsanguinated, often with an Esmarch bandage, to push blood into central circulation. Given the potential complications (highlighted in the literature) regarding deoxygenation with tourniquet use in SCD patients, this study focuses on assessing location and duration of tourniquet, three general complications (sickle cell crises, blood loss and transfusions, and increase of the mean arterial pressure); the seven following local potential risks were evaluated: phlebitis, skin complications, bone necrosis, muscle necrosis or abnormal muscle function, peripheral nerve impairment, elevated blood pressure, post-operative.

### Patients

The records of 233 SCD patients who underwent orthopaedic surgery with a tourniquet between 1978 and 2018 were retrospectively reviewed. These patients were followed by the same surgical and haematologic team with a mean 15 years follow-up (range 6 to 30 years) allowing a long-term assessment of the possible effect of the tourniquet. This study group (233 patients) was compared to a control group of 574 sickle cell patients followed by the same surgical team in the same hospital undergoing the same procedures in the same period between 1978 and 2018 but without a tourniquet.

### Study group with tourniquet: SCD anaemia group (233 patients)

#### Demographic

The patient cohort (Table 1) consisted of 138 men and 95 women. Most patients (125 patients) were homozygous for the sickle cell gene (haemoglobin SS), with 73 patients having haemoglobin S/haemoglobin C and 35 having haemoglobin S associated with beta-thalassemia. The patients’ age at surgery was an average of 32 years (14 to 55 years).

**Table 1:** Demographics Data

Demographic Parameter	Tourniquet Group	Non-Tourniquet Group
Women	95	234
Haemoglobin SS	125	308
Haemoglobin S/C	73	180
Haemoglobin S + Beta-Thalassemia	35	86
Average Age (years)	32	32
Age Range (years)	14–55	14–55

#### Surgery and tourniquet indication

- For lower limb (171 cases), indications were total knee arthroplasty for knee osteoarthritis (10 cases) or rheumatoid arthritis (12 cases) or knee osteonecrosis (20 cases), high tibial osteotomy of the proximal tibia for knee osteonecrosis (26 cases) or knee osteoarthritis (14 cases), knee arthroscopy (31 cases), ankle surgery for talus osteonecrosis (34 cases), ankle fracture (4 cases), tibia osteomyelitis sequelae (6 cases).
- For the upper limb (62 cases), indications were elbow osteonecrosis (7 cases), distal humerus osteomyelitis sequelae (4 cases), wrist arthroscopy (5 cases), carpal tunnel syndrome (22 cases), sequelae of Hand-Foot Syndrome (Dactylitis in 12 cases), carpal bone osteonecrosis (7 cases).

### Control group without tourniquet: SCD anaemia group (574 patients)

#### Demographic

The patient cohort (Table 1) consisted of 340 men and 234 women. Most patients (308) were homozygous for the sickle cell gene (haemoglobin SS), with 180 patients having haemoglobin S/haemoglobin C, and 86 having haemoglobin S associated with beta-thalassemia. The patient’s age at surgery was an average of 32 years (14 to 55 years).

#### Surgery indication

Lower limb (421 cases): Indications included total knee arthroplasty (TKA) for knee osteoarthritis (91 cases), rheumatoid arthritis (29 cases), or knee osteonecrosis (64 cases). High tibial osteotomy (HTO) was performed for knee osteonecrosis (124 cases) or knee osteoarthritis (34 cases). Knee arthroscopy was performed in 54 cases. Ankle surgery was indicated for talus osteonecrosis (19 cases), ankle fracture (2 cases), and tibia osteomyelitis sequelae (4 cases).

Upper limb (153 cases): Indications included elbow osteonecrosis (17 cases), distal humerus osteomyelitis sequelae (10 cases), wrist arthroscopy (12 cases), and carpal tunnel syndrome (54 cases). Additionally, hand-foot syndrome (dactylitis) was observed in 30 cases, while carpal bone osteonecrosis was present in 30 cases.

### Blood status and peri-operative management

Patients underwent a pre-operative evaluation, as previously described [4], including a haematologic consultation. Blood products, when necessary, were matched for ABO Rhesus (Cc D Ee) and Kell antigens to prevent alloimmunization. Antibiotics were administered during surgery and for three days afterward.

A specialized medical team monitored patients’ medical status and efforts to prevent complications experienced in the pre-operative management of patients undergoing orthopaedic procedures and managing medical complications. All patients underwent a pre-operative evaluation, including a haematologic consultation.

Among the 807 patients, 369 did not have a transfusion either pre-operatively or per-operatively or postoperatively: This concerns all upper limb procedures with or without tourniquet, knee arthroscopy, or ankle surgery.

On the other hand, the 438 patients who had surgery with tibial osteotomies and knee prostheses (considered as major procedures) had transfusion either with a tourniquet (42 TKA and 54 HTO) or without (184 TKA and 158 HTO). Transfusions were pre-operative (25 patients) or per-operatively for the others.

To prevent alloimmunization, given the antigen mismatch between primarily Caucasian donors and African-origin recipients, we have used phenotypically typed blood products for ABO, Rhesus (Cc; D; Ee), and Kell for the past 15 years. All patients were screened for antibodies before surgery. The national donor registry was utilized when needed.

Pre-operative red blood cell exchange was conducted for only 25 patients with a history of acute chest syndrome, prior cerebrovascular events, or severe anemia with haemoglobin levels below 5 g/dL, aiming to reduce haemoglobin S levels to under 30%. For other patients, simple acute transfusions were administered during surgery to maintain haemoglobin levels between 8 g/dL and 10 g/dL.

Oxygen saturation was monitored for three days and adapted with oxygen. Anticoagulants were administered postoperatively for a month for lower limb surgery.

### Tourniquet information

Knee, Foot and ankle, hand, and arthroscopic surgeons often request the application of a lower-limb or upper-limb tourniquet to create a bloodless field during surgery. However, there is a limit to how long a tourniquet can be applied, and where this tourniquet should be applied in SCD, proximal on the thigh or distal on the leg for the lower limb, and the same for the upper limb, proximal on the arm or distal on the forearm.

In this study, only a standard pneumatic tourniquet was proposed, either for the lower or upper limbs. The following data were collected: time of the tourniquet, position of the tourniquet proximally on the thigh or arm or distally on the forearm or leg. The pressure of the tourniquet and the mean blood pressure was measured during the procedure. Formal exsanguination of the limb with an Esmarch band was not used. Only the elevation of the limb during five minutes was used to decrease the amount of blood.

Delayed tourniquet inflation of around five minutes was done after intravenous antibiotic administration to allow circulation and appropriate antibiotic prophylaxis.

### Patient history, clinical examination before surgery, outcome clinical data

This study focused on the following eight potential risks: skin, arterial thrombosis, thrombo-phlebitis, neurological impairment of peripheral nerves, muscle necrosis or abnormal muscle function, bone necrosis, infection risk.

**Fig. 1:**
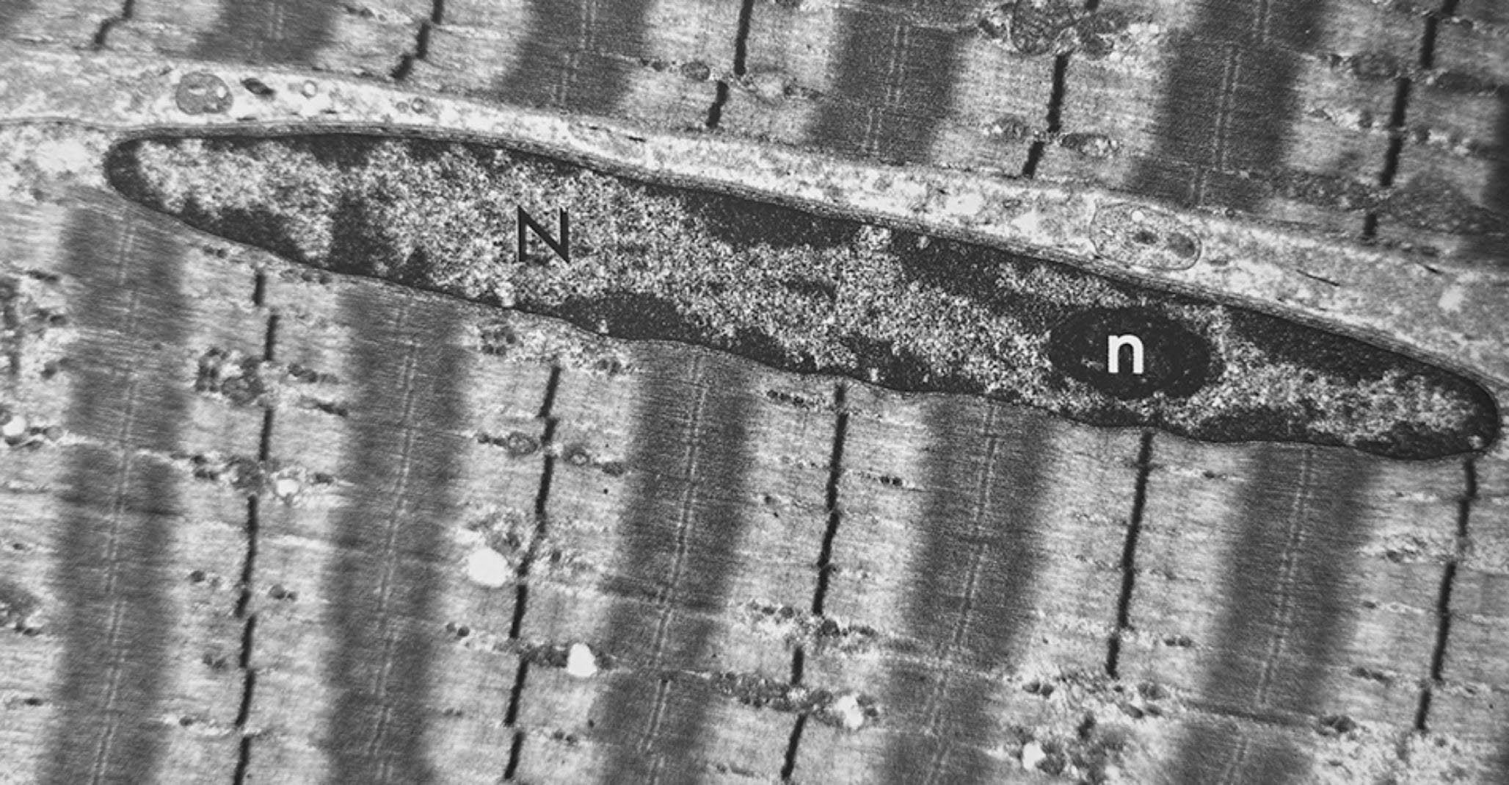
Normal muscle fiber: The nucleus (N) of the muscle fiber is located beneath the plasma membrane. It is very elongated and ovoid with the chromatin distributed towards the periphery. The nucleolus (n) is small and very dense (electron microscopy; EM; x13,000)

**Fig. 2:**
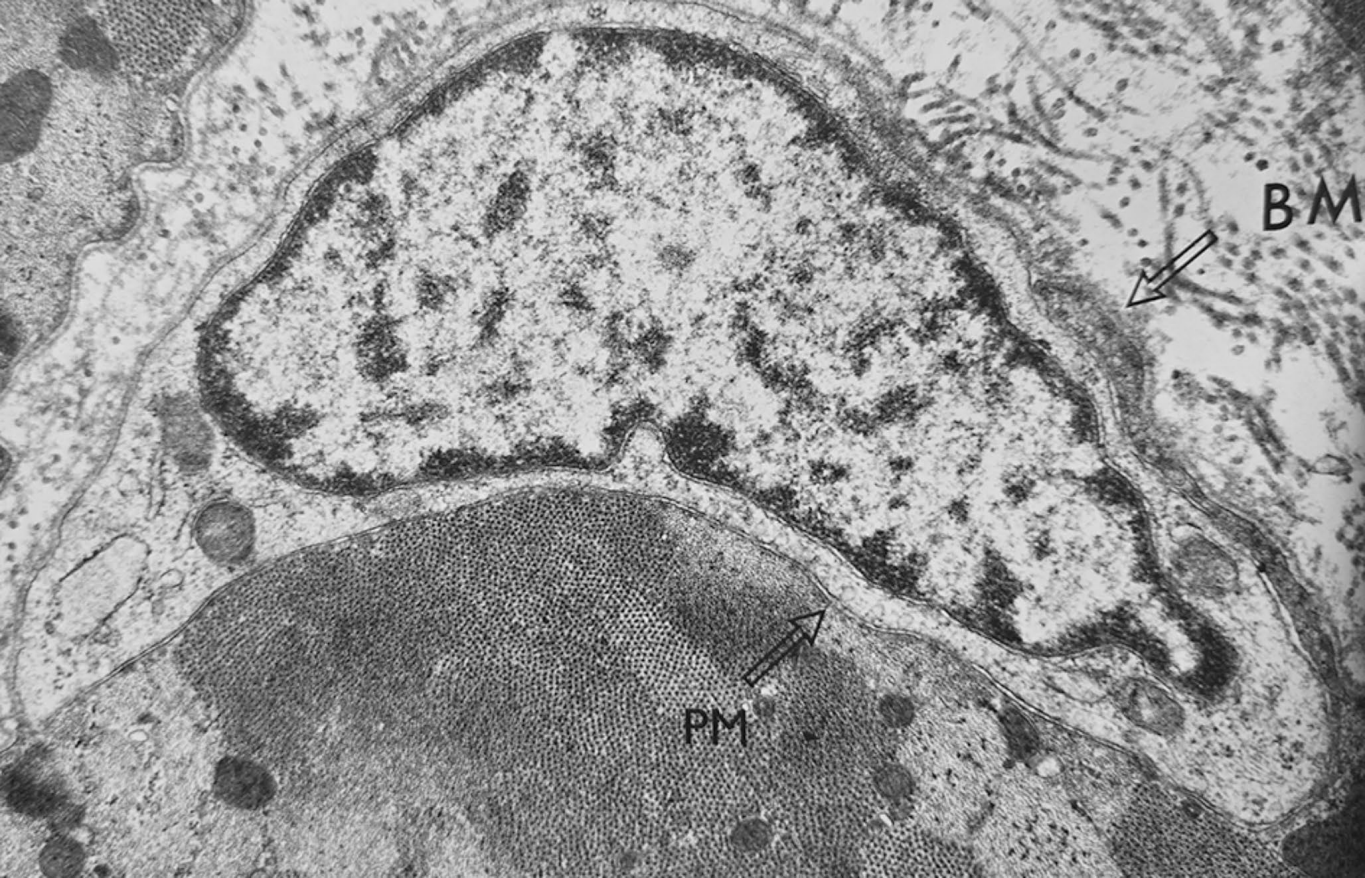
A satellite fibre is a mononuclear cell adjacent to a muscle fibre beneath the basement membrane (BM); It is located outside the fibre separated from it by the plasma membrane (PM) of each cell (EM x20,000). the nuclei of muscle fibres are normally located in the plasma membrane of the muscle fibre. The satellite cells on the side of the muscle fibres extend next to the basement membrane but are external to the plasmalemma and enclosed within their own cell membrane; these are mononuclear cells considered reparative stem cells

- The skin was examined pre-operatively and postoperatively. Leg ulcer prevalence was pre-operatively 8% in our population, with the same prevalence in the group control (no tourniquet) and in the study group with tourniquet. Wound healing, particularly for TKA and HTO, was rated with Southampton classification [6]. Prevalence of leg ulcer was analyzed at six years follow-up.
- Doppler ultrasonography was used to assess lower limb arterial health in patients with leg ulcer. Studies identified abnormal venous hemodynamic characteristics, but no presence of occlusive peripheral artery disease was found pre-operatively.
- Twelve patients had previous thrombophlebitis, meanly related to previous total hip arthroplasty [7]. Postoperatively, the independent association between tourniquet use and the cumulative 90-day incidence of VTE was predefined based on medical records documenting sonography-confirmed DVT or PE diagnosed through computed tomography or ventilation-perfusion scintigraphy. Therefore, VTE data was derived from a comprehensive review of clinical records.
- All patients underwent a neurological evaluation during the initial visit, with many presenting at least one abnormal neurologic finding. The study identified absent or hypoactive reflexes in numerous patients prior to surgery and tourniquet application. The most frequently observed abnormality was an impaired deep tendon reflex, affecting 7% of patients for the knee, followed by ankle (5%), biceps (4%), triceps (3%), ulnar (4%), and radial (3%) reflexes. Globally 8% of the patients had one or several impaired deep tendon reflex. The abnormality was bilateral in all the patients. Silent neurologic involvement may occur in SCD, but there is a possibility of subclinical involvement without neurologic signs and symptoms in some cases. When patients accepted, nerve electro-physiological examinations were proposed pre-operatively and postoperatively. Data were obtained in 14 patients in the study group with tourniquet and in 24 patients in the control group. Pre-operative peripheral nervous system involvement was detected in six patients with S/S genotype with sensorimotor axonal neuropathy.
- Muscles were analyzed for some patients with biopsies. Muscle biopsies were obtained in 12 patients with total knee arthroplasty. Biopsies from vastus lateralis muscle were obtained before the tourniquet pressure and at 30 min, 45 min, 60 min, 75 min, 90 min. They were analyzed with an optic and electronic microscope (Figs. 1 and 2). Muscle biopsies were also obtained after the 12 weeks with local anesthesia and was collected a few centimeters proximal from the first one, a small incision (10 mm) cutting superficial tissues was performed. A forceps was introduced in the subjacent muscle and the sample extracted (approximately 200 mg).

- Bones: Before surgery, all patients underwent an MRI, to look for osteonecrosis. Postoperatively, the same patients underwent an MRI, particularly in the event of new pain and distal osteonecrosis [8], which was focused on the site of the pain. Pre-operatively, the frequency was not different in patients with or without tourniquet.
- The infection risk was evaluated at 6 years followup in the two groups (with and without tourniquet).

### Statistics

Demographic properties of the patients and electrophysiological findings were summarized by descriptive statics. Chi-square test was used to determine the differences between the frequency of electrophysiological findings of the patient and control groups. Mann– Whitney U-test was conducted to identify the relationship between the frequency of pain crisis and the occurrence of peripheral neuropathy in patients with SCD.

Statistical significance was determined at *P* < 0.05. All statistical analyses were performed by SPSS version 11.0 software package.

## Results

In total, 807 SCD patients underwent surgical treatment (233 in the tourniquet group and 574 in the non-tourniquet group).

### Tourniquet data (all the patients)

#### Location of tourniquet

Tourniquet was proximal in the lower limb for 42 TKA, 54 HTO, for 31 knee arthroscopies, and for six osteomyelitis sequalae. It was only distal for 38 cases (22%). For upper limb (62 cases), it was proximal in 42 cases and distal in 20 cases.

#### Duration

Tourniquet inflation data was available for 233 cases, with a median duration of 65 min. Inflation lasted less than 30 min in 9% of procedures, while 42% had durations between 30 and 60 min, 45% between 61 and 90 min, and 5% exceeded 90 min.

### General complications (all the patients)

Medical complications were observed both in the tourniquet group and the non-Tourniquet group. Patients in the tourniquet group had the same time (*p* = 0.12) hospital stay versus the non-Tourniquet group (6.7 range 2 to 10 vs. 7.1, range 3 to 11 days).

### Painful sickling crises

86 patients had postoperatively painful sickling crises despite oxygen and intra operative transfusion for 48 patients. The prevalence of painful sickling crises was not different (*p* = 0.24) in the tourniquet and non-Tourniquet groups. On univariate logistic regression analysis, the transfused group had a lower (*p* = 0.04) prevalence of painful sickling crises than the non-transfused group.

### Blood loss and transfusions

Among the 438 patients who had transfusion during surgery, the Hb levels were similar between the groups pre-operatively and related to the genotype of the patient. During surgery, the total blood loss, which is determined by the Hb balance formula [9–10], with an approximation that a unit of banked blood generally contains approximately 52 ± 5.4 g of Hb [11]. The mean total blood loss in the tourniquet group (96 patients) was 438 ml (range 95 to 982), significantly lower (*p* = 0.031) than the 731 ml (range 246 to 1,376) observed in the non-tourniquet group (342 patients).

Despite per-operative transfusion, the non-tourniquet group had lower Hb on the third postoperative day. Post-operative transfusion was done when the level of hemoglobin dropped below 8 g/dL. Post-operatively More patients (120 patients) in non-tourniquet group required blood transfusions after surgery versus 20 patients in the tourniquet group, and this difference was statistically significant (*p* = 0.01).

Concerning transfusions, minor transfusions reactions were observed in 70 cases and were more frequent in the non-tourniquet group (40 cases). Despite the use of extended antigen-matched blood, major transfusion reactions occurred in 16 cases. Among these, 6 patients in the non-tourniquet group experienced massive intravascular hemolysis seven days post-transfusion, requiring a 10-day hospitalization in intensive care. Additionally, one patient developed acute chest syndrome.

### Mean arterial pressure

At 90 min, the mean arterial pressure in the proximal thigh group was 86.8 mmHg, compared to 76.3 mmHg in the distal leg group (*p* ≤ 0.014). By the end of surgery, this difference had further increased, with the proximal thigh group at 98.1 mmHg and the distal leg group at 78.3 mmHg (*p* ≤ 0.001). Shifting the site of compression during limb tourniquet application effectively prevents hypertensive responses. Therefore, when avoiding intraoperative hypertension is essential, employing a leg-tourniquet technique can mitigate this risk.

### Local complications and observations

#### Skin complications

Among these 807 patients, the most frequent complication was skin complications with skin necrosis at the tourniquet site, probably related to the tourniquet but also to the paint products. No difference skin wound healing grades, particularly in knee arthroplasty, was seen according to the Southampton Scoring System (*P* = 0.32). The prevalence of Leg ulcer at the most recent followup (mean 15 years follow-up; range 6 to 30 years) was 10% in our population, with the same prevalence in the control and study groups probably increased by the age of the patients when compared to the pre-operative evaluation (8%).

#### No arterial thrombosis was observed in each group

*Thrombophlebitis*

The cumulative 90-day incidence of deep thrombophlebitis (VTE) in lower limb was 30 (0.4%) with two pulmonary embolisms. There was no significant difference between the two groups, with nine VTEs occurring after a median of nine days in the tourniquet group compared to 21 VTEs after a median of 16 days in the no-tourniquet group (*P* = 0.08 for the cumulative 90-day VTE incidence). Similarly, the analysis examining the relationship between tourniquet duration and 90-day VTE incidence revealed no significant association, with 2 cases for tourniquet use under 30 min, 12 cases for 30–60 min, 14 cases for 61–90 min, and 2 cases for use exceeding 90 min (*P* = 0.4). Among these 30 cases, at the level of the upper limb, 3 phlebitis cases were diagnosed in patients without tourniquets.

*Neurologic observations*

Absence of deep tendon reflex was not increased after surgery both in the tourniquet and non-tourniquet group. Post-operative nerve electro physiologically examination demonstrated no change in the 38 patients who had this exam pre-operatively. Seven of these patients with tourniquet complained of abnormal residual pain after surgery with tourniquet; they had already had a pre-operative electromyogram showing abnormalities, but these abnormalities were not increased after surgery.

*Muscle observations and biopsy results (12 patients)*

In healthy individuals (Fig. 1) the nucleus (N) of the muscle fiber is located beneath the plasma membrane and is very elongated; the satellite cells (Fig. 2) are typically found beneath the basal lamina of myofibers, where they remain small in a quiescent state.

**Fig. 3:**
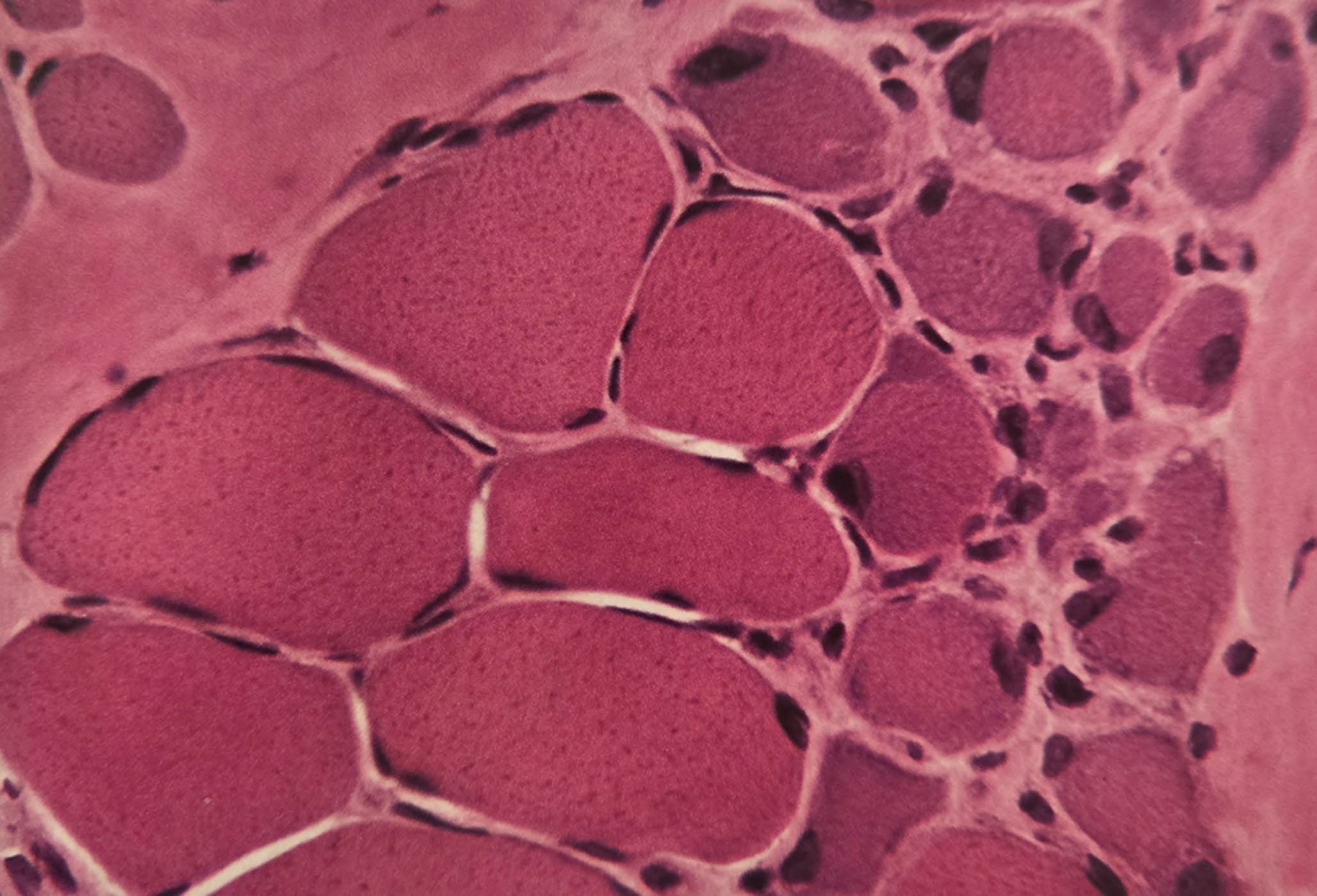
This group of small basophilic muscle fibers with large nuclei indicate a regeneration process (H&E, x600)

Before inflating the tourniquet, anomalies of skeletal muscle were observed in both groups (with and without tourniquet) of SCD patients. This remodeling was defined by the hypotrophy of type I and II muscle fibresand an increased satellite cell pool compared to healthy individuals (Fig. 3).

**Fig. 4:**
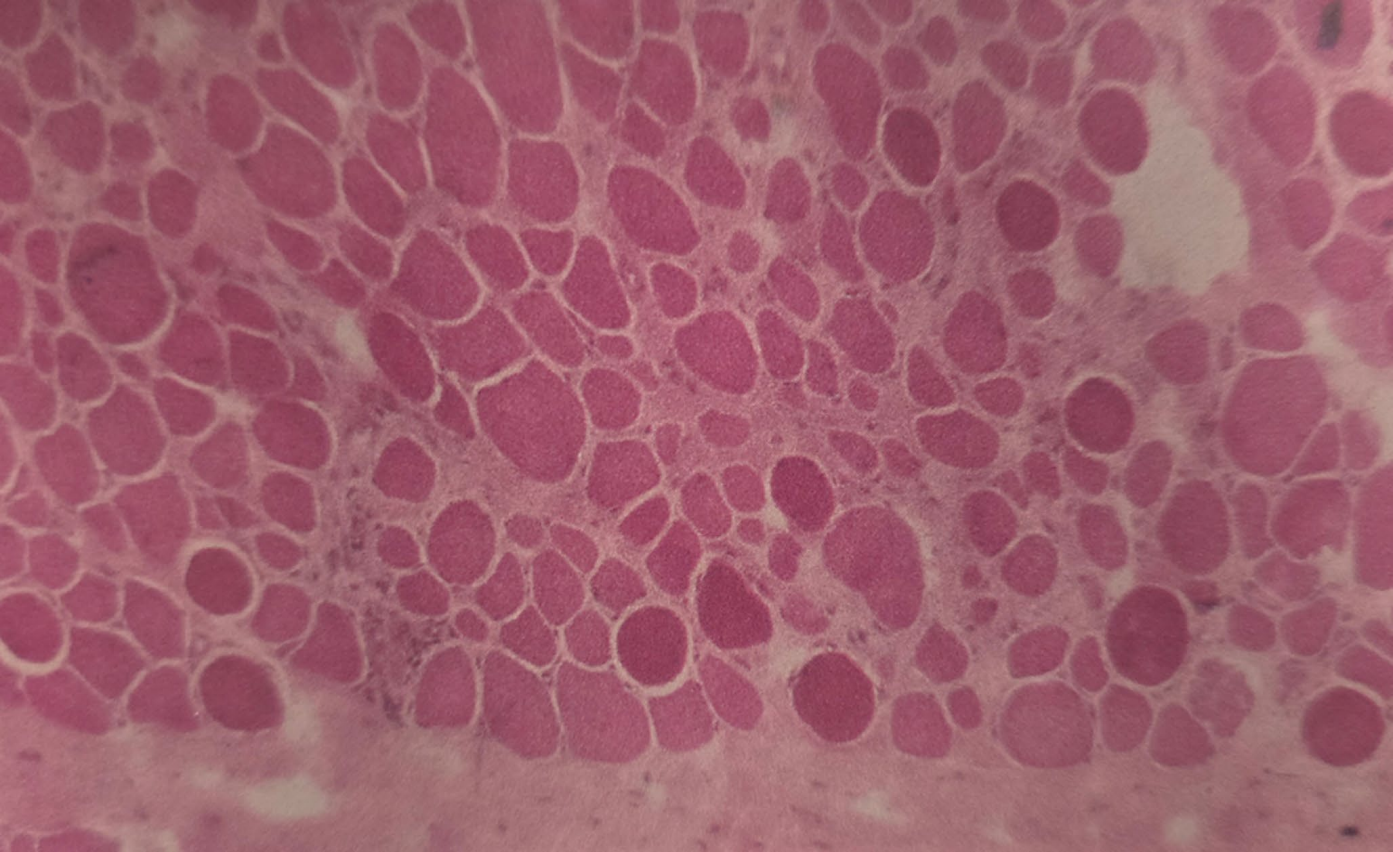
The wide variation in the size of muscle fibres, their rounded appearance and excess connective tissue indicate muscle distress (H&E, x 200)

**Fig. 5:**
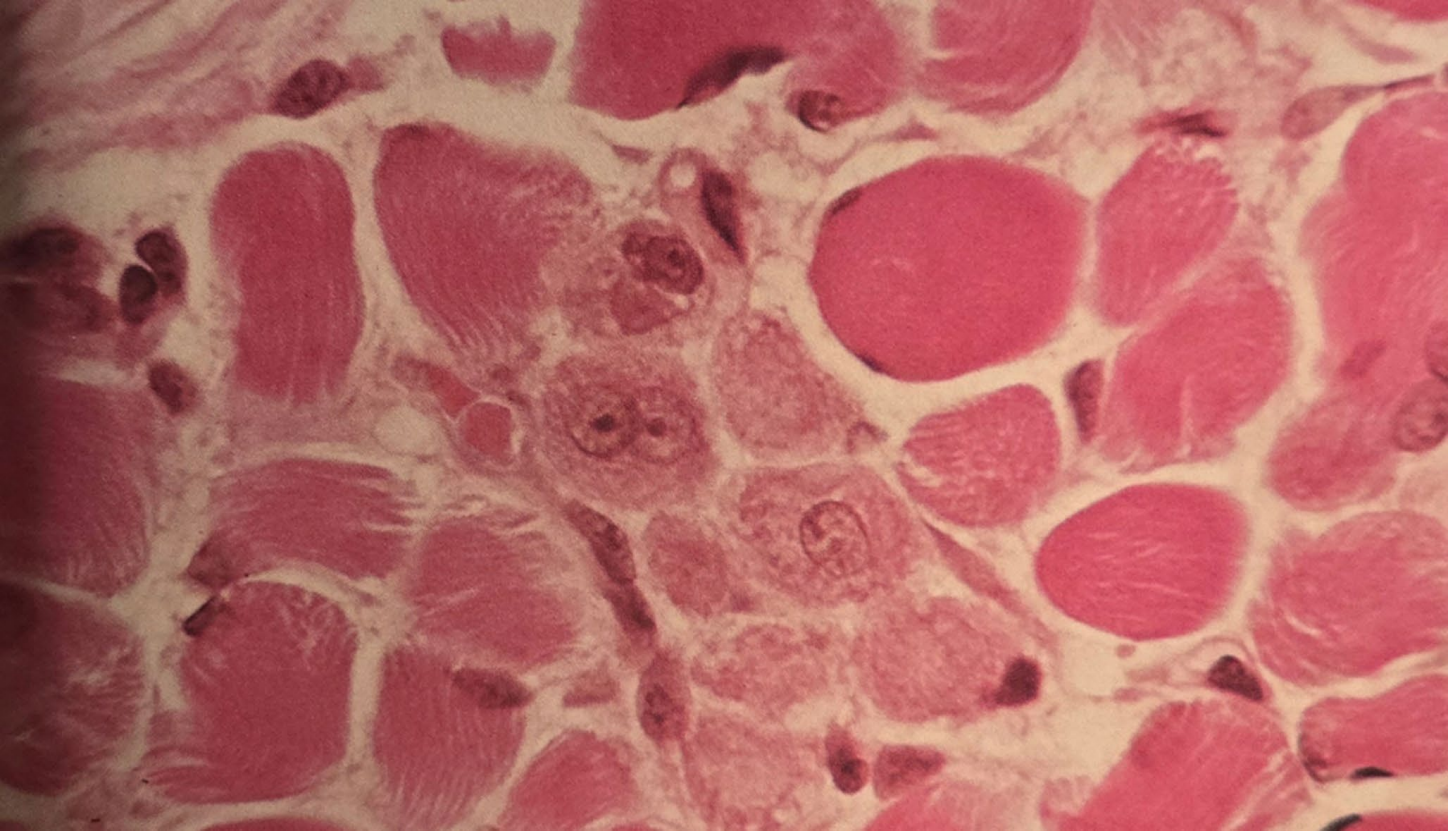
Regenerating fibres showing loose cytoplasm and large pale nuclei with prominent nucleoli (H&E, x300). Regardless of necrosis’s cause, the debris are removed by macrophages or phagocytic cells. Regeneration is initiated by the differentiation of satellite cells, which are normally found between the plasma and the basement membrane of muscle fibers. These mononuclear cells are considered to be the repair cells or stem cells. Their high RNA content (basophilia) and large vesicular nuclei identify the regenerating muscle fibres

No necrotic fibres were detected in any patient at any time in patients without tourniquet. Biopsies obtained under the tourniquet pressure detected no necrosis even after 90 min. However, muscle biopsies obtained after the 12-week period demonstrated some necrosis in patients with tourniquet (Fig. 4). No necrosis was observed in the non-tourniquet group. At the 12-week period patients in both groups (with and without tourniquet) displayed regenerative fibers and increase in volume of skeletal muscle satellite cells (Fig. 5).

*Bone*

30 new knee osteonecrosis cases and 15 new talus necrosis cases were diagnosed within three years after surgery; MRI confirmed their diagnoses. The frequency of occurrence of new cases was not different in patients with or without tourniquet.

*- The infection risk* was evaluated at six years followup in the two groups (with and without tourniquet). Two patients developed acute periprosthetic joint infections at four and five years post-surgery in the non-tourniquet group. No patient developed infection in the tourniquet group.

### Analysis of results

The prevalence of clinical complications was not different in the tourniquet and non-Tourniquet groups. On univariate logistic regression analysis, the tourniquet group had a lower prevalence of clinically important complications than the non-tourniquet group (*p* < 0.01). Univariate analysis revealed that in patients with complications in comparison with those without complications, those with complications were older at surgery entry (*p* < 0.001), had more painful crises (*p* < 0.001), and had more frequently the genotype S/S. Furthermore, they were more likely to have a pre-operative history of the following conditions: aseptic necrosis (*p* < 0.001), eye disease (*p* < 0.001), childhood osteomyelitis (*p* < 0.001), and hand-foot syndrome (*p* = 0.004). They were also more likely to have undergone blood transfusion (65.4% vs. 53.7%, *p* < 0.001). Laboratory findings indicated that patients with complications exhibited higher reticulocyte counts (*p* = 0.025) and lower levels of haemoglobin F (Hgb F, *p* = 0.005) and albumin (*p* < 0.001).

## Discussion

Not all sickle cell patients are the same, just as not all tourniquets are. Moreover, not all countries have access to the same technology or benefit from the same level of expertise; more specifically, some countries have no access to oxygen or blood transfusions.

In this study, we will compare sickle cell anaemia patients undergoing surgery with and without the use of a tourniquet. The first key finding of this study highlights the frequency of pre-existing abnormalities in the nerves and muscles of sickle cell patients, independent of any surgical intervention or tourniquet use. The second significant finding is the lack of a meaningful difference in the incidence of complications between patients operated on with a tourniquet and those operated on without one, provided the surgery is performed in a centre experienced in managing sickle cell patients. The 3rd important point is that the use of tourniquets in sickle cell patients must be the result of an analysis of the benefits and risks in these patients.

Regardless of the patient or the surgical procedure, using a tourniquet in a healthy patient [15] and in a sickle cell patient always carries some risk, no matter how small [12, 13, 14, 15]. This risk is further heightened in patients known to have skin-related complications, independent of the type of orthopaedic surgery being performed. Additionally, histological abnormalities in muscle tissue and were well-documented in this patient population, even in absence of clinical consequences.

While it is clear that tourniquet use presents a potential hazard, it is equally important to consider the challenges posed by blood loss during surgery and the advantage of the tourniquet in saving blood, particularly in TKA and HTO [16]. This can be a significant issue in sickle cell patients, particularly given the difficulty of securing compatible blood for transfusion. This challenge is even more pronounced in many underdeveloped regions, where access to safe and compatible blood supplies is limited. The outcomes of different previous studies and case reports are not uniform. The interventions differed in tourniquet type, peri-operative care, and operative procedure with a very small number of cases in each study. Furthermore, in some regions, pneumatic tourniquets are often unavailable or non-functional. As a result, alternative methods are commonly employed, such as the Esmarch band, or other historical techniques [17, 18, 19]. These realities highlight the need for a balanced approach, weighing the risks and benefits of tourniquet use while considering the resources available and the specific needs of sickle cell patients.

However, patients with tourniquet under general anaesthesia have a progressive elevation in blood pressure which may be an inconvenient in SCD. The hypertension appears to be linear over time and can require attempts to control or reduce it. Unfortunately, the blood pressure elevation is unresponsive to many drugs, including potent opioids. Aside from other concerns regarding tissue ischemia injury, these physiological changes limit the duration a tourniquet can be applied, even in general anesthesia in SCD. The exact mechanism of tourniquet-induced hypertension is unknown, and certain occurrences are difficult to explain. Awake patients in upper limb [20] with no general anaesthesia report discomfort and for the lower limb a dull ache in the whole of the limb that worsens with time, typically becoming unbearable at 20 min with some hypertension. Regardless of the exact mechanism of action by which the hypertension is generated, the “line of crush” (tissue compressed by the inflated tourniquet) is probably the key trigger. This study showed that moving the location of the tourniquet to a more distal location on the leg can reduce the mean arterial pressure and subsequently the hypertensive response. But this is not possible in many surgeries.

Because this study highlights the frequency of pre-existing abnormalities in the nerves of sickle cell patients, it may be helpful to image nerves [21–22] in these patients before using a tourniquet.

According to our measurements out comes, the study provides critical insights into the safety of using tourniquets in sickle cell disease (SCD) patients undergoing orthopaedic surgeries. Despite initial concerns about exacerbating sickling complications, the data suggests that, when properly managed, tourniquets can be used safely. There was no major difference in the rate of painful sickling crises, infection, deep vein thrombosis (DVT), or neurological complications between the tourniquet and non-tourniquet groups. The tourniquet group had significantly lower intraoperative blood loss (438 ml vs. 731 ml, *p* = 0.031), which led to fewer post-operative transfusions (20 vs. 120 patients, *p* = 0.01). This is a crucial benefit, as blood transfusions in SCD patients carry risks such as alloimmunization and transfusion reactions. The incidence of new osteonecrosis and infections did not differ significantly between groups.

Based on the findings, several recommendations can be made to optimize the use of tourniquets in SCD patients while minimizing risks: Tourniquet use should be considered on a case-by-case basis, particularly in patients with pre-existing vascular complications or high risk of thrombosis. Patients with a history of severe anemia or acute chest syndrome should be evaluated for preoperative red blood cell exchange to reduce haemoglobin S levels below 30%. Continuous oxygen monitoring and supplementation should be used to prevent hypoxia, which can trigger sickling.

For intraoperative strategies, proximal placement (thigh or upper arm) was associated with a higher mean arterial pressure compared to distal placement (leg or forearm), suggesting a need to consider more distal placement when possible. Tourniquet duration should ideally be limited to less than 90 min.

For the postoperative management, the cumulative incidence of VTE was low (0.4%) and similar between groups. However, anticoagulation for at least one month postoperatively remains crucial, particularly for lower limb surgeries.

Overall, the study suggests that tourniquet use is a viable option in SCD patients when managed with careful patient selection, intraoperative monitoring, and postoperative follow-up. The decision should be individualized, weighing the benefits of reduced blood loss with the other risks.

In conclusion, despite the limitations of this study, particularly its single-rcentre design in an experienced facility and the absence of pediatric patients, it provides valuable insights into the use of tourniquets in sickle cell disease.

## Data Availability

No datasets were generated or analysed during the current study.
